# Case report: Intra-atrial course of right coronary artery: Evaluation by dual-source CT

**DOI:** 10.4103/0971-3026.76057

**Published:** 2011

**Authors:** Johann Christopher, Chary Duraikannu

**Affiliations:** Department of Cardiology, CARE Hospital, Hyderabad, India; 1 Department of Radiology, CARE Hospital, Hyderabad, India

**Keywords:** Dual-source CT, intra-atrial, right coronary artery

## Abstract

We report a case of an anomalous course of the right coronary artery (RCA) through the right atrium, which was evaluated using dual-source CT angiography. There have been a few cases described previously in literature, but most of them were found either during surgery or at autopsy. Although this variant is clinically benign, it has significant consequences during interventional procedures or bypass surgery.

## Introduction

Intra-atrial or intracavitary course of the right coronary artery (RCA) is a rare anomaly (incidence of 0.09–0.1%),[1] with a clinically benign outcome. Previously, this anomaly was found either during surgery or at autopsy. With the advent of coronary CT angiography, this condition is now being detected prospectively. Awareness of its presence may help prevent major catastrophes during surgery or interventional procedures.[1–6]

## Case Report

A 48-year-old postmenopausal female presented with complaints of chest pain, dyspnea, and palpitation. There were no risk factors, and she had no previous cardiac interventions or surgery. The treadmill test was mildly positive, and she was advised to undergo coronary CT angiography.

CT coronary angiography was performed using a SOMATOM Definition (Siemens, Germany) dual-source CT scanner. The total calcium score was zero. The following parameters were used during the CT angiography study: 120/120 kV; 171 mAs/rotation; rotation time, 330 ms; and pitch, 0.2; 64 × 0.6 mm collimation. Using a dual-head injector (Injektron CT2; Medtron, Saarbrucken, Germany), 70 ml of nonionic iohexol (Omnipaque 350, GE Healthcare, Cork, Ireland) was administered by bolus tracking with 30 ml of saline. The total scan time was 12.2 s. The total CT radiation dose, expressed as DLP (dose-length product), was 504 mGy/cm. Reconstruction was done with 0.6-mm slice thickness and 0.3-mm intervals, using a medium-smooth convolution kernel in the most optimal diastolic and systolic phases.

**Figure 1 (A-D):**
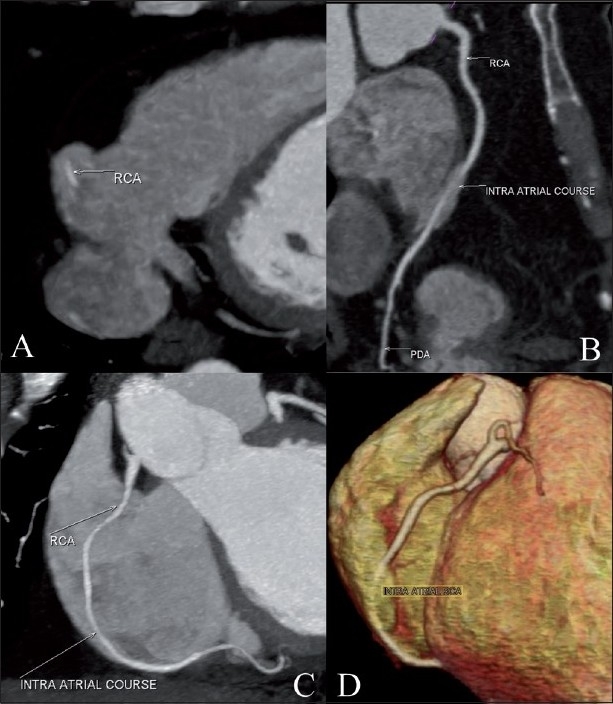
Cardiac CT. Axial maximum intensity projection (MIP) image (A) shows the intra-atrial location of the right coronary artery (RCA) (white arrow). The curved multiplanar reconstruction (B) and MIP (C) images show the normal origin and short intra-atrial course of the RCA (white arrows). The volume-rendered image (D) shows the entry and exit points of the RCA. Also, note the normal epicardial course of the proximal and distal RCA

**Figure 2 (A,B):**
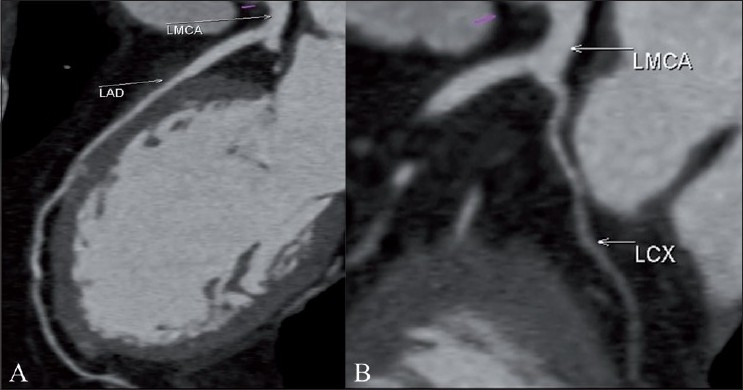
Cardiac CT. The curved MPR images show the origin and course of the left main coronary (LMCA), left anterior descending (LAD) (A), and left circumflex artery (LCX) (B)

CT coronary angiography showed normal origins of the right and left main coronary arteries. The proximal RCA had a normal epicardial course between the pulmonary trunk and the right atrial appendage. The mid-RCA was seen to penetrate the right atrial wall inferolaterally and after a short (1.5 cm) intra-atrial course, exited the right atrium inferiorly. The distal RCA had an epicardial course and it divided into the posterolateral ventricular branch (PLVB) and the posterior descending artery (PDA) at the posterior interventricular groove [Figure 1 A–D]. The rest of the coronary arteries, including the left main coronary, the left anterior descending, and the left circumflex (as well as its branches), were normal in course [Figures 2 A–B]. There was no evidence of coronary artery plaques.

## Discussion

The RCA arises from the anterior coronary sinus. It normally has an epicardial course anteroinferiorly in the right atrioventricular groove and continues around the margin of the heart toward the crux, where it divides into PDA and PLVB.

In our case, the origin of the artery and the proximal RCA were normal, but the mid-RCA had a short intra-atrial course of 1.5 cm. Previous case reports of intra-atrial RCA evaluated by CT angiography have shown variable lengths of 2.5 to 5 cm.[2,3] After the intra- atrial course, the distal RCA emerged into the epicardium proximal to the crux. There was no evidence of narrowing either at the site of entry or at the exit of the RCA in the atrial wall. The entire RCA, including the PDA and the PLVB, was normal in caliber. Although the patient’s symptoms cannot be attributed to this anomalous course of the RCA, it is of great significance, especially when an interventional procedure is planned. There may be a greater risk of damage to the RCA during indirect cannulation of the inferior vena cava or coronary sinus, during invasive electrophysiological procedures, and during radiofrequency ablation of atrial tachyarrhythmias arising in the right atrium. This may result in distal myocardial ischemia or a left-to-right shunt during the procedure. Also, during coronary artery bypass surgery, inadvertent entry into the right atrium can lead to suction of air into the right atrium, causing an air lock of the cardiopulmonary bypass circuit.[2,3,6]

There are three abnormal locations of the coronary arteries, which include aerial, mural, and intracavitary.[4] The aerial location does not cause much of a problem during invasive angiography or bypass grafting. But the other two are significant variants with respect to interventional procedures. An intracavitary RCA could be disrupted, especially during pacemaker implantation, right heart catheterization, or invasive electrophysiology testing.[5] Similarly, it can complicate coronary artery bypass surgery, leading to difficulties in vessel localization or bypass grafting.

The detection of intracavitary/intra-atrial RCA would be difficult with invasive coronary angiography. Multidetector CT coronary angiography is however a good technique to identify this anomaly. Identifying this anomalous course beforehand provides crucial information to the interventional cardiologist or surgeon.
